# Identification of a *De Novo* Heterozygous Missense *ACTB* Variant in Baraitser–Winter Cerebrofrontofacial Syndrome

**DOI:** 10.3389/fgene.2022.828120

**Published:** 2022-03-24

**Authors:** Kailai Nie, Junting Huang, Longqian Liu, Hongbin Lv, Danian Chen, Wei Fan

**Affiliations:** ^1^ Department of Ophthalmology, West China Hospital, Sichuan University, Chengdu, China; ^2^ Research Laboratory of Ophthalmology and Vision Sciences, State Key Laboratory of Biotherapy, West China Hospital, Sichuan University, Chengdu, China; ^3^ Department of Ophthalmology, The Affiliated Hospital of Southwest Medical University, Luzhou, China

**Keywords:** Baraitser–Winter cerebrofrontofacial syndrome, ACTB gene, missense variant, *de novo* variant, targeted gene sequencing

## Abstract

Baraitser–Winter cerebrofrontofacial syndrome (BWCFF, OMIM: 243310) is a rare autosomal-dominant developmental disorder associated with variants in the genes *ACTB* or *ACTG1*. It is characterized by brain malformations, a distinctive facial appearance, ocular coloboma, and intellectual disability. However, the phenotypes of BWCFF are heterogenous, and its molecular pathogenesis has not been fully elucidated. In the present study, we conducted detailed clinical examinations on a Chinese patient with BWCFF and found novel ocular manifestations including pseudoduplication of the optic disc and nystagmus. Targeted gene panel sequencing and Sanger sequencing identified a *de novo* heterozygous missense c.478A > G (p.Thr160Ala) variant in *ACTB*. The mRNA and protein expression of *ACTB* was assessed by quantitative reverse transcription PCR and Western blots. Furthermore, the functional effects of the pathogenic variant were analyzed by protein structure analysis, which indicated that the variant may affect the active site for ATP hydrolysis by the actin ATPase, resulting in abnormal filamentous actin organization in peripheral blood mononuclear cells. This discovery extends the *ACTB* variant spectrum, which will improve genetic counseling and diagnosis, and may contribute to understanding the pathogenic mechanisms of actin-related diseases.

## Introduction

Baraitser–Winter cerebrofrontofacial syndrome (BWCFF, OMIM: 243310) is a rare congenital developmental disorder characterized by facial anomalies, coloboma, short stature, and intellectual disability (Baraitser and Winter 1988). Distinctive facial features include hypertelorism, ptosis, high-arched eyebrows, and a broad nasal tip and bridge. Predominant abnormalities in the structure of the nervous system, including pachygyria and lissencephaly, are associated with seizures, sensorineural deafness, and intellectual disability (Di Donato et al., 2014; Verloes et al., 2015; Yates et al., 2017). A previous study suggested that the pathogenic variants in the actin genes *ACTB* and *ACTG1* cause BWCFF (Rivière et al., 2012). Although both *ACTB* and *ACTG1* are ubiquitously expressed throughout the body and share very similar amino acid sequences, they possess distinct biological properties; thus, the clinical phenotype of BWCFF caused by variants in *ACTB* and *ACTG1* are not identical (Perrin et al., 2010; Verloes et al., 2015; Yates et al., 2017). Currently, approximately 100 cases have been reported in the literature. However, the relationships between the *ACTB* and *ACTG1* variants and the resultant different BWCFF phenotypes have not been fully elucidated. The correlations between the genotypes and phenotypes of BWCFF in the Chinese population are not well-documented. In this study, we identified a *de novo* heterozygous missense *ACTB* variant *via* targeted gene panel sequencing and Sanger sequencing in a Chinese pedigree with BWCFF. We conducted comprehensive ophthalmic imaging of the proband, and combined with functional analysis of the variant, evaluated its effect on ACTB. Our results may help in understanding the potential molecular mechanism underlying this syndrome.

## Materials and Methods

### Clinical Assessment

The pedigree consisted of a proband and two family members, medical histories were taken, and all participants underwent height, weight, and finger length measurements. Routine laboratory assessments were carried out, including analyses of complete blood cell count, coagulation function, liver function, and kidney function. Detailed ophthalmic examinations were performed including slit lamp microscopy, optometry examination, fundus photography, ultrasound, and wide-field retinal imaging with fundus fluorescence photography under general anesthesia (RetCam 3 system, Natus) (She et al., 2021). The proband was further evaluated *via* cranial magnetic resonance imaging (MRI), chest x-ray, echocardiogram, and abdomen ultrasound scan. The Wechsler Preschool and Primary Scale of Intelligence (WPPSI, IV edition) was used to assess the patient’s cognitive development, and growth information since birth was collected from the kids’ health recording chart.

### Targeted Gene Panel Sequencing

Targeted next-generation sequencing with a gene panel including 663 disease-related genes (Supplementary Material, Table 1) was performed in this pedigree, according to the protocols as previously described in our study (Huang et al., 2019). Blood samples of the family members were collected, and genomic DNA was isolated using a standard extraction method. Qualified genomic DNA was randomly fragmented into 200-bp segments. After end repair, A-tailing, adaptor ligation, and PCR amplification, the products were validated using an Agilent 2100 Bioanalyzer (Agilent). The target gene regions were enriched according to the manufacturer’s protocols (MyGenostics), and the captured libraries were sequenced on an HiSeq 2000 platform (Illumina). Clean reads were aligned to the reference human genome hg19 using the Burrows–Wheeler Aligner. Single-nucleotide polymorphisms (SNPs) and insertion–deletion mutations were detected and annotated using GATK and ANNOVAR. Identified variants were filtered according to minor allele frequency <0.5% using the ExAC and 1000 Genomes Project database, and disease-causing variants reported in the Human Gene Mutation Database (HGMD) and ClinVar database were considered (Zhang et al., 2020).

**TABLE 1:** PCR and Sanger sequencing primers (5′–3′).

Primer name	Sequence	Primer name	Sequence
*actb478-F*	TTA​ATG​TCA​CGC​ACG​ATT​TCC	*actb478-R*	ACT​TAG​CCG​TGT​TCT​TTG​CAC
*ACTB-F*	ACA​GAG​CCT​CGC​CTT​TGC​C	*ACTB-R*	GAT​ATC​ATC​ATC​CAT​GGT​GAG​CTG​G
*ACTG1-F*	CGC​AAG​TAC​TCG​GTG​TGG​AT	*ACTG1-R*	TGC​TAC​GCA​TCT​GCT​GAG​TC
*GAPDH-F*	TGA​GAA​CGG​GAA​GCT​TGT​CA	*GAPDH-R*	GCA​AAT​GAG​CCC​CAG​CCT​TC

### Sanger Sequencing and Cosegregation Analysis

Primers for the *ACTB* NM_001,101.5 c.478A > G variant were designed using the NCBI Primer-Blast tool (Table 1). Genomic DNA samples of the pedigree and a group of 100 healthy controls (Huang et al., 2019) were PCR-amplified and purified. The PCR products were sequenced on an ABI3500 Genetic Analyzer (Applied Biosystems), and then cosegregation analyses of the pedigree were performed based on the sequencing results (Fu et al., 2020).

### Assessment of the Effect on ACTB Protein

The effects of amino acid substitution on the ACTB protein were predicted using the programs, including REVEL, MutationTaster, FATHMM, and SIFT. Multiple protein sequence alignment and BLAST (Basic Local Alignment Search Tool) of ACTB and homologs from several representative eukaryotes were performed using the NCBI HomoloGene database (https://www.ncbi.nlm.nih.gov/homologene/110648). A *Homo sapiens* ACTB protein structure with 2.50-Å resolution was obtained from the RCSB Protein Data Bank (PDB ID: 6NBW) (Rebowski et al., 2020). Then homology modeling analysis of the p.Thr160Ala variant was carried out using the SWISS-MODEL (Waterhouse et al., 2018) based on this monomeric ACTB structure, and the QMEANDisCo score corresponding to the modeled protein structure quality was confirmed.

### Quantitative Reverse Transcription PCR

Peripheral blood mononuclear cells (PBMCs) from the proband and healthy controls were isolated according to the Histopaque-1077 manual (Sigma Aldrich). Total RNA extraction from PBMC and reverse transcription were performed using TRIzol Reagent (Invitrogen) and PrimeScript RT Kit with gDNA Eraser (TaKaRa), respectively. Primer pairs of ACTB-F/R, ACTG1-F/R, and GAPDH-F/R (Table 1) were used for quantitative PCR with the aid of TB-Green Taq Mix (TaKaRa), and *GAPDH* served as the reference gene. Each experiment was performed in three replicate wells and repeated three times; multiple comparisons were conducted by ANOVA. Data are presented as mean ± standard deviation (SD).

### Western Blot

PBMCs were rinsed twice with PBS and collected by centrifugation, then lysed on ice in RIPA lysis buffer supplemented with protease inhibitor cocktail (Beyotime). After determining the protein concentration using the BCA protein assay kit (Beyotime), the appropriate sample solution volumes were calculated and mixed with the loading buffer (Bio-Rad). Proteins were separated *via* electrophoresis using 10% SDS-polyacrylamide gels (SDS-PAGE) and transferred to 0.2-µm PVDF membranes (Bio-Rad). The membranes were incubated overnight at 4°C with the following primary antibodies: rabbit antiACTB (1:50,000, AC026, ABclonal) and mouse antiGAPDH (1:20,000, 60004-1-Ig, Proteintech). Afterward, corresponding secondary antibodies including IRDye 680RD goat anti-mouse and IRDye 800CW goat anti-rabbit (1:15,000, Li-Cor) were incubated for 1 h at room temperature. Protein blots were detected using the Odyssey CLx system (Li-Cor).

### Immunofluorescence

PBMCs from the proband and healthy controls were cultured for 7 days in RPMI 1640 medium with 10% fetal bovine serum (Gibco) on glass cover slips coated with Matrigel (Corning). Cells were fixed with 4% paraformaldehyde and permeabilized with 0.1% Triton X-100, rinsed with PBS, and blocked in 1% bovine serum albumin. Rabbit anti-TUBA1B (α‐Tubulin, 1:200, 11224-1-AP, Proteintech) primary antibodies were applied for overnight incubation. Alexa Fluor-conjugated secondary antibodies (1:500, Abcam) and FITC-conjugated phalloidin (1:50, Beyotime) to label filamentous actin (F-actin) were incubated for 1 h in the dark (Rivière et al., 2012). Coverslips were mounted with antifade reagent with DAPI (Invitrogen) and visualized using a fluorescent microscope (Nikon). Finally, imaging analysis was carried out using the ImageJ software (*n* = 20 cells in each group).

## Results

### Clinical Characteristics

**FIGURE 1:**
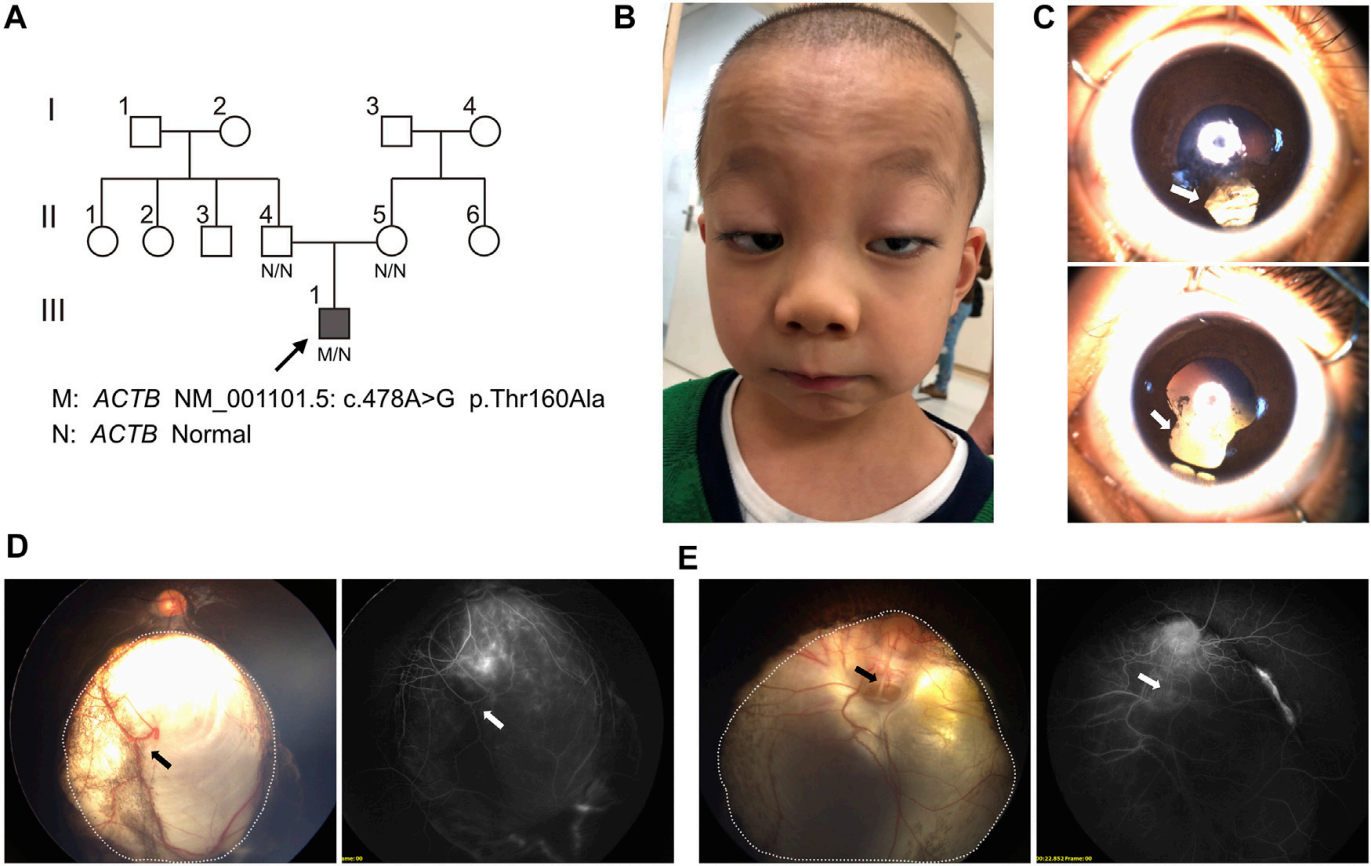
Pedigree and facial, ophthalmological characteristics. **(A)** Pedigree and genotype of proband (III.3, arrow, filled square) and unaffected individuals, which were marked as blank symbols (circles, female; squares, male). **(B)** Facial anomalies present ptosis, highly arched eyebrow, epicanthal fold, hypertelorism, wide nasal bridge, anteverted nares, long philtrum, thin upper lip, and retrognathia. **(C)** Bilateral iris coloboma (arrow) with iris pigment adhesion to the anterior lens capsule. **(D)** Wide-field fundus imaging and fluorescence angiography showed the chorioretinal coloboma (the area within the dotted line) and large choroidal vessel (arrow) in the right eye. **(E)** Chorioretinal coloboma (the area within the dotted line) were indicated in the left eye and a pseudoduplication of the optic disc below the real optic disc; angiography revealed vessels radiating from the defect (arrow).

**FIGURE 2:**
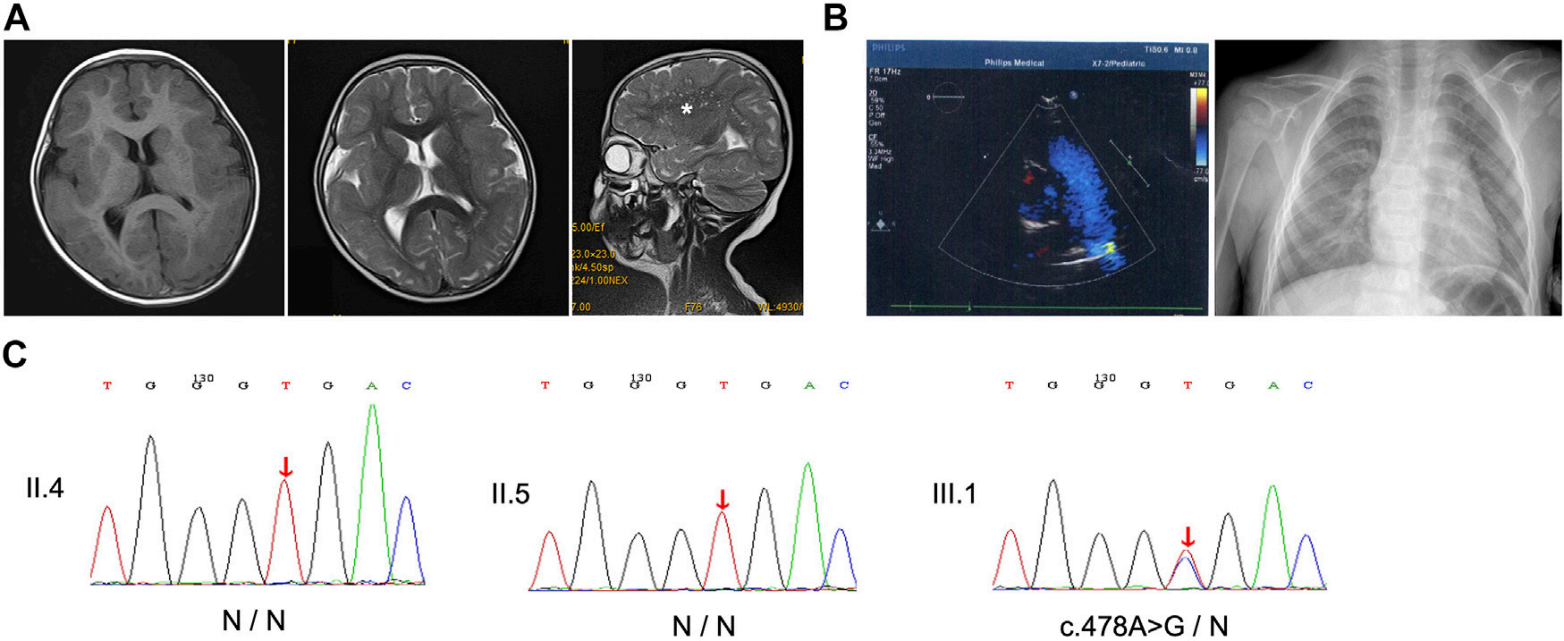
Imaging and Sanger sequencing. **(A)** Cranial T1- and T2-weighted magnetic resonance imaging (MRI) of the patient at age 3 showed that pachygyria was more pronounced in bilateral frontal lobes, and sagittal T2-weighted image revealed enlarged perivascular spaces (asterisk). **(B)** Echocardiogram and chest x-ray exclude heart defects. **(C)** Sanger sequencing presents the heterozygous missense c.478A > G variant in the proband, while his parents did not reveal the presence of the mutation. Red arrows, variant sites.

The proband was a 6-year-old boy who was the first born of his healthy nonconsanguineous parents with a full-term uneventful gestation (Figure 1A,III.1). Physical examinations revealed distinct facial features, including ptosis, highly arched eyebrow, epicanthal fold, hypertelorism, wide nasal bridge, anteverted nares, long philtrum, thin upper lip, and retrognathia (Figure 1B). The boy had a short neck with a head circumference of 47.9 cm (3rd centile), a mild short stature with a height of 107 cm (3rd centile), and weight of 17.6 kg (3rd–10th centile) (Pu et al., 2021). Ophthalmologic examinations showed congenital binocular horizontal nystagmus, iris coloboma with iris pigment adhesion to the anterior lens capsule (Figure 1C), and chorioretinal coloboma and pseudoduplication of the optic disc in the left eye (Figures 1D, E). Optometry examination indicated right eye −0.50 and left eye −3.00 diopter of spherical refractive error. Cranial MRI indicated pachygyria and enlarged perivascular spaces (Figure 2A), while chest x-ray, echocardiogram, and abdomen ultrasound scans did not reveal anomalies. The results of routine blood counts indicated no thrombocytopenia (platelet count 263 × 10^9^/L), and coagulation function was normal. He had intellectual developmental delay and attention deficit; and was not able to complete the WPPSI test due to poor cooperation. He started walking at age 3, can only speak a few words (e.g. “papa” and “mama”) since then, and is now able to do simple things, such as eating and playing with toys independently. He can respond to his name being called but cannot cooperate with hearing tests. The patient underwent umbilical hernia surgery at the age of 1 year old and had suffered recurrent seizures since the age of 3 years old. The seizures manifest as partial limb clonus with consciousness; the duration was within 30 min, and the frequency varied from once a day to twice a month. The seizures were not treated with medication, and the proband’s parents refused a referral to the neurology clinic.

### Pathogenic Variant Screening

Targeted next-generation sequencing for the proband and his parents identified a missense variant c.478A > G in exon 4 of *ACTB* (NM_001,101.5), resulting in an amino acid substitution of threonine (Thr) to alanine (Ala) at position 160 (p.Thr160Ala). While the heterozygous variant was verified by Sanger sequencing in the proband, it was absent in both parents who showed no disease phenotype (Figure 2). Parentage was confirmed by the trio analysis of hundreds of variants in common between the proband and his parents, and the variant was considered to be *de novo* in the proband. Moreover, the c.478A > G variant was absent in controls according to Sanger sequencing. Searches in the 1000 Genomes Project, ExAC, and HGMD databases suggested that the variant was novel. Taken together, these findings suggest that the variant is potentially pathogenic.

### Analysis of Conservation of Amino Acids

**FIGURE 3:**
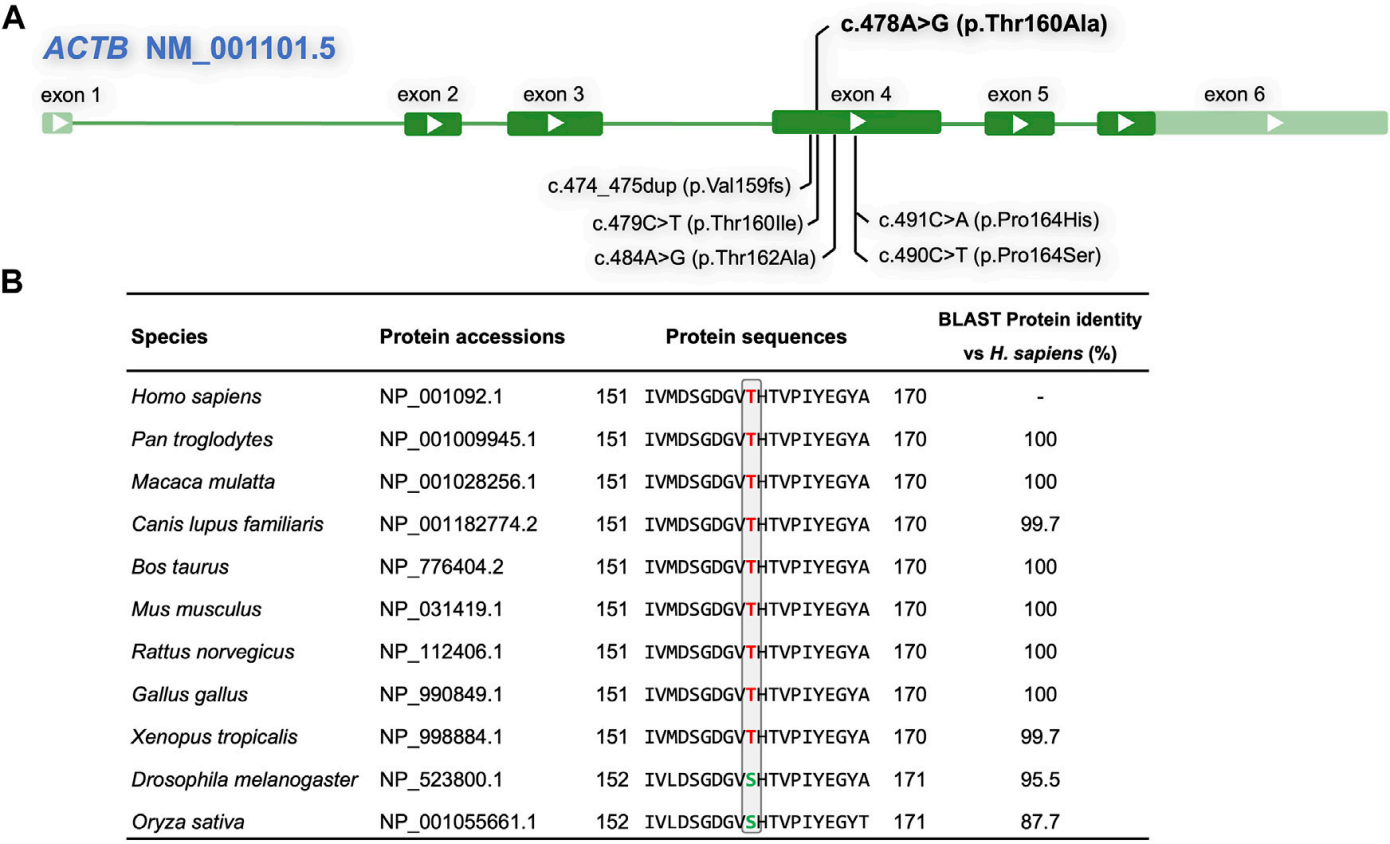
Outline of the *ACTB* gene and variants, and conservation analysis. **(A)** Compilation of the novel p.Thr160Ala and adjacent *ACTB* variants reported in the literature and the ClinVar database associated with BWCFF. **(B)** Multiple alignments showing amino acid conservation at the mutant sites across the indicated species; red letters indicate conserved threonine, whereas green letters indicate serine, which is a hydrophilic amino acid with a hydroxyl group on the side chain like threonine.

The commonly used algorithms REVEL, MutationTaster, FATHMM, and SIFT predicted that the c.478A > G p.Thr160Ala (Figure 3A) variant was damaging. The predicted disease-causing effects are summarized in Table 2. Analyses of homolog alignment revealed that threonine at site 160 is highly conserved in many species (Figure 3B), particularly in species above amphibians (*X. tropicalis*) with a full-length protein sequence identity greater than 99.7%. In *Drosophila* and rice (*O. sativa*), that site is occupied by serine, which, like threonine, is also a hydrophilic amino acid with a hydroxyl group on the side chain. Hence, the replacement of threonine by the hydrophobic alanine in the p.Thr160Ala variant may distort the local structure of the ACTB protein.

**TABLE 2:** Characteristics of ACTB variant and analysis of disease-causing effects.

Gene	Exon	Variation			REVEL	MutationTaster	FATHMM	SIFT
		Nucleotide	Protein	Alleles				
*ACTB*	4	c.478A > G	p.Thr160Ala	Het	Disease causing (0.704)	Disease causing (0.99)	Damaging (−4.85)	Damaging (0.02)

Note. c, coding DNA, reference sequence; p, protein reference sequence; Het, heterozygous; REVEL, rare exome variant ensemble learner; FATHMM, functional analysis through hidden Markov models; SIFT, sorting intolerant from tolerant.

### Protein Modelling

**FIGURE 4:**
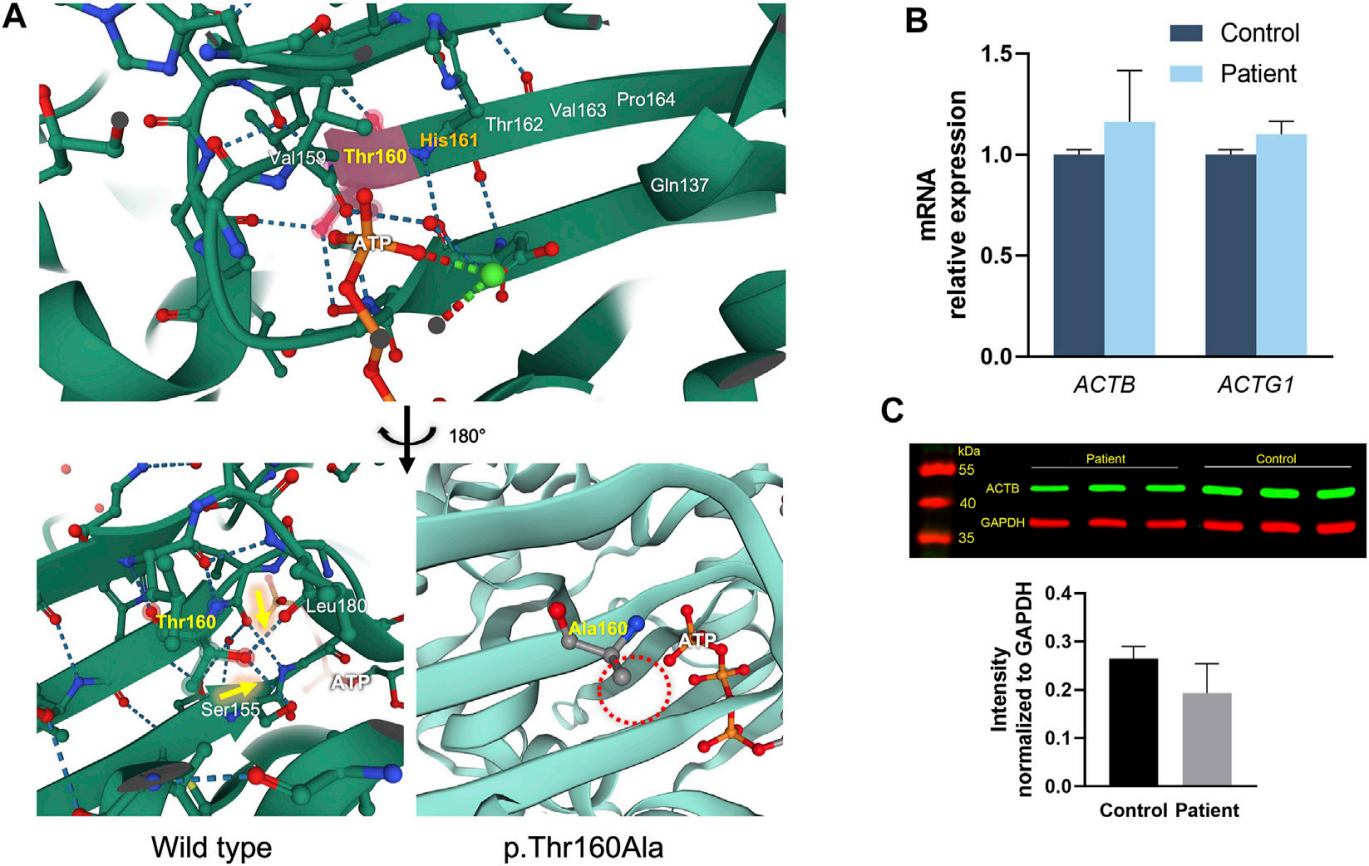
ACTB protein structure and variant modeling, and expression analysis. **(A)** Thr160 is close to His161, which is the catalytic base of ATP hydrolysis. The hydroxyl group side chain of Thr160 forms hydrogen bonds with the spatially close Leu180 and Ser155 (yellow arrows), and the p.Thr160Ala variant results in alanine without a hydroxyl group in the side chain, which cannot form hydrogen bonds with the surrounding residues (dashed red circle). **(B)** mRNA expressions of *ACTB* and *ACTG1* in the PBMCs of the patient and healthy controls. **(C)** Western blot showing that the variant does not change the ACTB protein expression in PBMCs (*p* > 0.05); GAPDH was used as loading control.

ACTB protein structure analysis revealed that Thr160 is close to His161, which is the catalytic base of ATP hydrolysis (Figure 4A) (Merino et al., 2018; Chou and Pollard, 2019; Rebowski et al., 2020). Moreover, Thr160 is the beginning of the *ß*-fold, and the hydroxyl group of its side chain forms hydrogen bonds with the spatially closed Leu180 and Ser155, suggesting that Thr160 is involved in maintaining the local conformation. Residue variant modeling of p.Thr160Ala showed that alanine, without the hydroxyl group in the side chain, could not form hydrogen bonds with the surrounding residues.

### RNA and Protein Expression

The RNA expressions of *ACTB* and *ACTG1* in PBMCs isolated from the proband and normal controls were assessed (Figure 4B). We found that the c.478A > G variant did not affect *ACTB* mRNA or lead to altered expression of *ACTG1.* Western blot likewise showed that the p.Thr160Ala variant did not change the amounts of ACTB protein in the PBMCs of the patient (Figure 4C).

### Cellular Morphology

**FIGURE 5:**
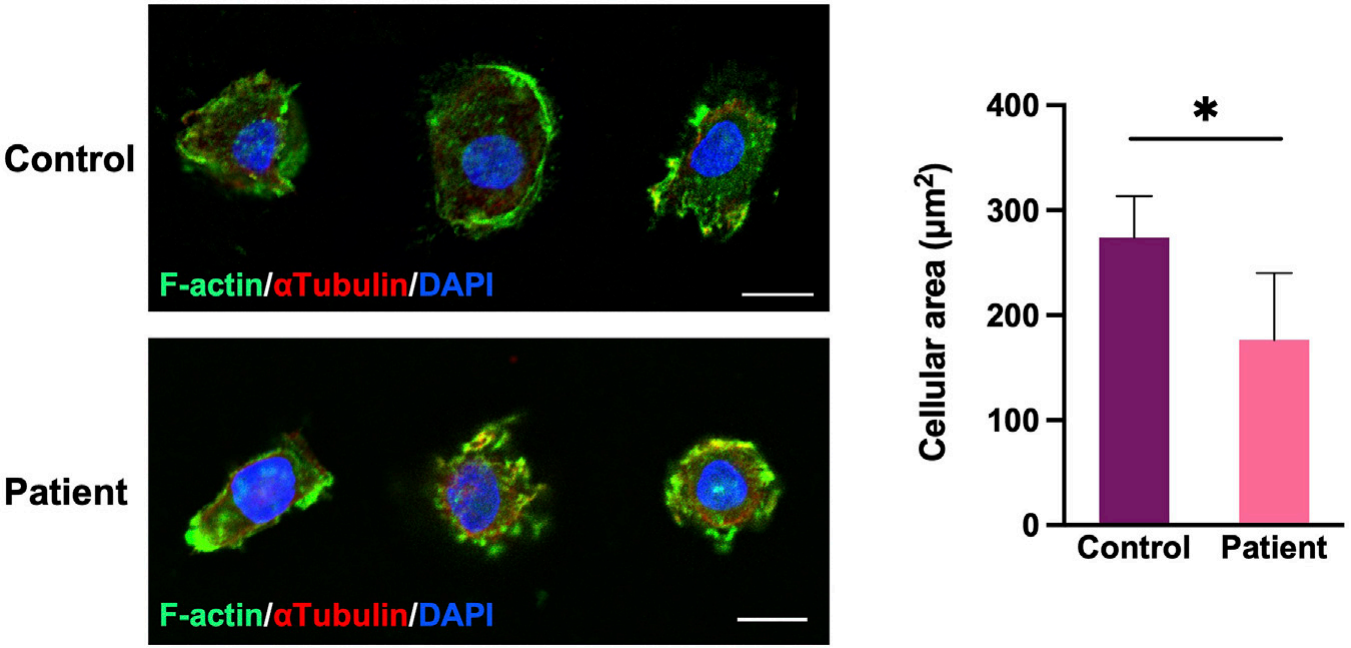
Cell morphology and filamentous actin (F-actin) of the cytoskeleton of PBMCs. PBMCs double labeled for F-actin (green) and *a*-tubulin (red). The patient’s PBMCs extended into a smaller range, resulting in a significant decrease in the area of a single PBMC (*n* = 20 cells in each group; *p < 0.05, two-tailed *t test*). Scale bar, 10 μm.

By comparing the morphologies and the filamentous actin (F-actin) of the cytoskeleton of PBMCs between the proband and healthy controls, we observed that the patient’s PBMCs extended into smaller range under the same culture condition, resulting in a significant decrease in the single cellular area (*p* < 0.05, Figure 5). This suggests that the variant may alter the cytoskeleton organization of F-actin.

## Discussion

BWCFF is a very rare genetic disease associated with ACTB variants. In previous case reports, the phenotype of each patient was heterogeneous with different genetic variants, which posed some challenges for clinical diagnosis (Rivière et al., 2012; Johnston et al., 2013; Di Donato et al., 2014; Verloes et al., 2015; Yates et al., 2017; Latham et al., 2018; Sandestig et al., 2018; Sun et al., 2018; Baumann et al., 2020; Chacon-Camacho et al., 2020; Hampshire et al., 2020). In this study, the patient showed distinct BWCFF facial and neurodevelopmental anomalies and ocular coloboma. Using targeted gene sequencing and Sanger sequencing, we identified a novel *de novo* heterozygous missense *ACTB* variant c.478A > G (p.Thr160Ala) in the patient. Analysis of amino acid conservation, protein structure, gene expression, and cellular function suggested that the variant is pathogenic in accordance with the American College of Medical Genetics and Genomics guidelines (Richards et al., 2015).

We first presented wide-field fundus imaging and fluorescence angiography of a patient with BWCFF. Typical features of chorioretinal coloboma and a rare pseudoduplication of the optic disc were found. Furthermore, detailed ophthalmologic examinations indicated that the patient had a congenital binocular horizontal nystagmus, which has rarely been reported in previous studies. However, nystagmus is not a characteristic manifestation of a particular disease; it may be secondary to several neurological disorders. In this patient, the onset of nystagmus was likely associated with retinal and central nervous system dysplasia. In addition to the typical ocular manifestations of BWCFF, there are a number of less commonly reported ocular phenotypes that physicians should keep in mind. Rall et al. (2018) reported an unusual stroma of irises and corectopia in a patient with a heterozygous *ACTB* c.1092_1105dup14 (p.Ile369SerfsX18) variant. The p.Ala58Val variant in *ACTG1* reportedly led to right esotropia in a three-generation pedigree with BWCFF (Kemerley et al., 2017). Two cases of sporadic BWCFF patients with congenital strabismus were recently reported, and the heterozygous *ACTG1* pathogenic variants p.Gln59Arg and p.Thr203Met were identified in these patients, respectively (Chacon-Camacho et al., 2020). In a patient with a p.Gln59Arg variant, congenital extraocular muscle fibrosis was confirmed during surgery.

The actin monomer polymerizes to form F-actin accompanied by the binding of ATP, and then actin hydrolyzes ATP and releases phosphate. This is the key process of actin filament assembly and disassembly, and the fundamental mechanism of both cytoskeleton assembly and cellular functions (Merino et al., 2018; Chou and Pollard, 2019; Rebowski et al., 2020). Our data indicate that the p.Thr160Ala variant is located near the active site of ATP hydrolysis, His161, and it affected the hydrogen bond between the side chain of the Thr160 and the surrounding Leu180 and Ser155 residues. This may impair the stability of the spatial structure and, thus, the ATP hydrolysis activity of the catalytic site His161. Hence, the alteration in the amino acid residue may lead to changes in the rate of hydrolysis of ATP, which, in turn, may affect cytoskeleton assembly and cellular function. Hundt et al. (2014) analyzed the actin assembly behavior of a p.Arg183Trp variant of ACTB, which is close to the ATP nucleotide-binding pocket of actin. Compared with wild-type actin, the ATP hydrolysis in p.Arg183Trp actin was increased, polymerization was slower, and depolymerization was faster, which compromised the formation of stable filaments. In a cellular morphology assay of PBMCs, we observed a reduction in the area of single PBMC in patient vs. controls. This may be related to the altered F-actin assembly caused by the variant (Procaccio et al., 2006). Mutations in actin also lead to inappropriate migration of neuronal cells, which are associated with pachygyria, agenesis of the corpus callosum, and enlarged ventricles (Vontell et al., 2019).

Sun et al. (2018) reported a BWCFF patient with an ACTB p.Thr162Ala, which was also close to His161 (Figure 3A). This patient had clinical features of facial abnormalities, pachygyria, and mild dysplasia in the corpus callosum, similar to our case. However, that patient suffered more severe epilepsy, and his detailed ocular phenotype was not described. There are several variants located close to His161 in the ClinVar database: p.Val159fs (VCV001319337), p.Thr160Ile (VCV000372917), p.Thr162Ala (VCV001202044), and p.Pro164His (VCV000973260). Both of them purportedly involve a condition of BWCFF and interpretated as likely pathogenic or pathogenic, but those submitters did not provide more detailed information. Some cases with *ACTB* gene alteration but without BWCFF phenotype have been recently described (Cuvertino et al., 2017; Latham et al., 2018; Baumann et al., 2020). The cohort of patients generally carried deletion, frameshift, and nonsense variants in the *ACTB* gene, which were considered as loss-of-function variants leading to haploinsufficiency. Specifically, several variants in exons 5 and 6 of ACTB were associated with syndromic thrombocytopenia (Latham et al., 2018).The c.478A > G (p.Thr160Ala) variant in this study was a heterozygous missense variant located in exon 4, and the patient showed a typical BWCFF phenotype, several routine blood tests did not indicate platelet count reduction. In addition, we found that the expressions of *ACTB* mRNA and protein were not significantly altered in patients compared with normal controls. This is consistent with the previously reported p.Arg183Trp (Procaccio et al., 2006), p.Arg196His (Rivière et al., 2012), and p.Gly302Ala (Baumann et al., 2020) variants. Thus, we infer that the variant caused the disorder by gain‐of‐function or dominant‐negative mechanisms (Rivière et al., 2012; Di Donato et al., 2014; Verloes et al., 2015; Baumann et al., 2020). The correlation between *ACTB* genotype and phenotype, and the underlying pathogenesis needs to be further determined.

In conclusion, we identified a *de novo* heterozygous missense c.478A > G (p.Thr160Ala) variant in *ACTB*, which caused BWCFF in the patient and demonstrated detailed ophthalmologic manifestation. In addition, gene expression analysis, protein modeling, and *in vitro* cellular functional studies suggested that the variant may influence cytoskeletal organization by affecting actin ATPase activity. Thus, our findings extend the ACTB variant spectrum and may help reveal the potential mechanisms of the *ACTB* variant leading to BWCFF.

## Data Availability

The datasets presented in this study can be found in online repositories. The names of the repository/repositories and accession number(s) can be found in the article/Supplementary Material.
