# Supplementary material for: Identification of a De Novo Heterozygous Missense ACTB Variant in Baraitser–Winter Cerebrofrontofacial Syndrome
Source: Front Genet. 2022 Mar 24;13:828120. doi: 10.3389/fgene.2022.828120 (PMC8989421; doi:10.3389/fgene.2022.828120)
Supplement: Supplementary file 1 [file Table1.DOCX]

**Supplementary Table 1.** List of genes screened in this study.

| ABCA4 | ADAM9 | ADIPOR1 | ALDH1A3 | ARL2BP | ATOH7 | BBIP1 | BBS9 |
| --- | --- | --- | --- | --- | --- | --- | --- |
| C10orf11 | C5AR2 | CACNA2D4 | CDH23 | CERKL | CHM | CLN5 | CNGB1 |
| COL4A1 | CRB1 | CRYBB2 | CST3 | CYP1B1 | DMD | DTHD1 | EMD |
| ERCC5 | FAM126A | FKTN | FRMD7 | GALT | GJB1 | GNPTG | GSTM1 |
| HDAC6 | HMCN1 | HSF4 | IFT172 | IQCB1 | KCNV2 | KLC2 | LCA5 |
| LRAT | MAF | MERTK | MLPH | MT-ND4 | MTTP | MYOT | NEK2 |
| NPHP1 | NXNL1 | OPA3 | OR2W3 | PAX3 | PDE6D | PEX13 | PEX5L |
| PIKFYVE | PNPLA6 | PPT1 | PRPF4 | RAB27A | RAX | RDH12 | RLBP1 |
| RP9 | SAG | SEC23A | SHOX | SLC25A15 | SLC4A11 | SNRNP200 | SRD5A3 |
| TBC1D24 | TCTN3 | TGFBR2 | TMEM114 | TMEM98 | TRNT1 | TTR | TYRP1 |
| VAX1 | WDPCP | ZEB1 | ABCB6 | ADAMTS10 | AGBL1 | ALMS1 | ARL3 |
| ATP13A2 | BBS1 | BCOR | C12orf57 | C5orf42 | CAPN3 | CDH3 | CFB |
| CHMP4B | CLN6 | CNGB3 | COL6A1 | CRX | CRYBB3 | CTC1 | CYP27A1 |
| DMPK | DTNBP1 | EPG5 | ERCC6 | FAM161A | FLVCR1 | FSCN2 | GCNT2 |
| GJB2 | GP1BA | GSTT1 | HESX1 | HMGB3 | HTRA1 | IFT27 | IRX5 |
| KCTD7 | KLHL7 | LCAT | LRIT3 | MAK | MFN2 | MPDZ | MT-ND4L |
| MVK | NAA10 | NEUROD1 | NPHP3 | NYX | OPA6 | OTX2 | PAX6 |
| PDE6G | PEX14 | PEX6 | PITPNM3 | POC1B | PRCD | PRPF6 | RAB28 |
| RAX2 | RDH5 | RNASEH1 | RPE65 | SALL1 | SELENON | SIL1 | SLC25A4 |
| SLC6A5 | SOD1 | SREBF2 | TBK1 | TDRD7 | TIMM8A | TMEM126A | TMX3 |
| TRPM1 | TUB | UBIAD1 | VCAN | WDR19 | ZNF408 | ABCC6 | ADAMTS17 |
| AGBL5 | ANO5 | ARL6 | ATP1A3 | BBS10 | BEST1 | C12orf65 | C8orf37 |
| CAPN5 | CDHR1 | CFH | CHN1 | CLN8 | CNNM4 | COL6A2 | CRYAA |
| CRYGA | CTDP1 | CYP4V2 | DNA2 | DUX4 | EPHA1 | ERCC8 | FBLN5 |
| FOXC1 | FTL | GDF3 | GLI2 | GPR143 | GUCA1A | HEXA | HMX1 |
| IARS2 | IGBP1 | ITGA2B | KERA | KMT2D | LCT | LRP5 | MAN2B1 |
| MFRP | MPV17 | MT-ND5 | MYH2 | NAT2 | NF1 | NPHP4 | OAT |
| OPN1LW | P3H2 | PCDH15 | PDE6H | PEX16 | PEX7 | PITX2 | POLG |
| PRDM13 | PRPF8 | RAB3GAP1 | RB1 | RECQL4 | RNLS | RPGR | SALL2 |
| SEMA4A | SIX5 | SLC26A4 | SLC7A14 | SORD | STRA6 | TBX1 | TEAD1 |
| TIMP3 | TMEM138 | TNNT1 | TSPAN12 | TUBA8 | UCHL1 | VHL | WDR36 |
| ZNF423 | ABHD12 | ADAMTS18 | AGK | ANTXR1 | ARMS2 | ATP2C1 | BBS12 |
| BFSP1 | C1QTNF5 | C9 | CASK | CEP164 | CFHR1 | CHST6 | CLRN1 |
| CNOT9 | COL6A3 | CRYAB | CRYGB | CTNNA1 | CYP51A1 | DNAJC5 | DYSF |
| EPHA2 | ESR1 | FBN1 | FOXD3 | FTO | GDF6 | GMPPB | GPR179 |
| GUCA1B | HEXB | HPS1 | IDH3B | IMPDH1 | ITGA7 | KIAA1549 | KRT12 |
| LGR4 | LRPAP1 | MANBA | MFSD8 | MRE11 | MT-ND6 | MYH7 | NBAS |
| NF2 | NR2E1 | OCA2 | OPN1MW | P3H3 | PCYT1A | PDZD7 | PEX19 |
| PGK1 | PITX3 | POLG2 | PRIMPOL | PRPH2 | RAB3GAP2 | RBP3 | RGR |
| ROBO3 | RPGRIP1 | SBF2 | SGCA | SIX6 | SLC2A1 | SLC9A6 | SOX10 |
| SYNE1 | TCAP | TENM3 | TINF2 | TMEM216 | TOPORS | TTC21B | TUBB3 |
| UNC119 | VIM | WDR73 | ZNF469 | ACBD5 | ADAMTSL4 | AGPS | AP3B1 |
| ASB10 | ATXN10 | BBS2 | BFSP2 | C2 | CA4 | CAV1 | CEP250 |
| CFHR3 | CIB2 | CLUAP1 | COL11A1 | COL7A1 | CRYBA1 | CRYGC | CTNND2 |
| DCN | DNM1L | EFEMP1 | ERBB3 | EXOSC2 | FBN2 | FOXE3 | FXN |
| GFER | GNAT1 | GRK1 | GUCY2D | HFE | HPS3 | IDO1 | IMPG1 |
| ITGB3 | KIF11 | KRT3 | LIM2 | LTBP2 | MAPKAPK3 | MIP | MTHFR |
| MT-TI | MYH9 | NDP | NHS | NR2E3 | OCRL | OPN1MW2 | P3H4 |
| PDCD2 | PEX1 | PEX2 | PGR | PLA2G5 | POMGNT1 | PRKCG | PRPS1 |
| RAB7A | RBP4 | RGS9 | ROM1 | RPGRIP1L | SC5D | SGCB | SLC16A12 |
| SLC33A1 | SLITRK6 | SOX2 | SYNE2 | TCF4 | TFAP2A | TK2 | TMEM231 |
| TPM2 | TTC8 | TUBGCP4 | UNC45B | VLDLR | WFS1 | ZNF513 | ACO2 |
| ADAR | AHI1 | APOE | ASRGL1 | ATXN7 | BBS4 | BLOC1S3 | C21orf2 |
| CABP4 | CAV3 | CEP290 | CFI | CISD2 | CNBP | COL11A2 | COL8A2 |
| CRYBA2 | CRYGD | CTSD | DHCR7 | DOCK9 | ELOVL4 | ERCC2 | EYA1 |
| FGFR3 | FOXL2 | FYCO1 | GJA1 | GNAT2 | GRM6 | GUSB | HGF |
| HPS4 | IDUA | IMPG2 | ITM2B | KIF21A | LAMA1 | LMNA | LYST |
| MAPT | MITF | MTM1 | MT-TL1 | MYO5A | NEB | NMNAT1 | NR2F1 |
| OFD1 | OPN1SW | PABPN1 | PDE6A | PEX10 | PEX26 | PHOX2A | PLEKHA1 |
| POMT1 | PROM1 | PRSS56 | RABGGTA | RCBTB1 | RGS9BP | RP1 | RRM2B |
| SCO2 | SGCD | SLC16A2 | SLC38A8 | SMOC1 | SPATA7 | TACSTD2 | TCOF1 |
| TGFB3 | TLR3 | TMEM237 | TPM3 | TTLL5 | TUBGCP6 | USH1C | VPS13B |
| WHRN | ZNF644 | ACTA1 | ADGRA3 | AIPL1 | APTX | ATF6 | B3GLCT |
| BBS5 | BLOC1S6 | C2orf71 | CACNA1A | CC2D2A | CEP41 | CFL2 | CLDN19 |
| CNGA1 | COL18A1 | COL9A1 | CRYBA4 | CRYGS | CTSF | DHDDS | DRAM2 |
| ELP4 | ERCC3 | EYS | FHL1 | FRAS1 | FZD4 | GJA3 | GNB3 |
| GRN | HARS | HGSNAT | HPS5 | IFNGR1 | INPP5E | JAG1 | KIF7 |
| LAMA2 | LMX1B | LZTFL1 | MC1R | MKKS | MT-ND1 | MT-TL2 | MYO7A |
| NECTIN1 | NOD2 | NRL | OLFM2 | OPTC | PANK2 | PDE6B | PEX11B |
| PEX3 | PHYH | PLG | POMT2 | PRPF3 | PXDN | RABGGTB | RD3 |
| RHO | RP1L1 | RS1 | SDCCAG8 | SGCG | SLC24A1 | SLC39A5 | SMS |
| SPG7 | TAT | TCTN1 | TGFBI | TLR4 | TMEM67 | TPP1 | TTN |
| TULP1 | USH1G | VSX1 | WRN | ACTB | ACTG1 | ALDH18A1 | ARL13B |
| ATM | B9D1 | BBS7 | BMP4 | C3 | CACNA1F | CCDC28B | CEP78 |
| CHD7 | CLN3 | CNGA3 | COL2A1 | COL9A2 | CRYBB1 | CSPP1 | CX3CR1 |
| DHX38 | DRD5 | EMC1 | ERCC4 | EZR | FKRP | FREM2 | GALK1 |
| GJA8 | GNPAT | GSN | HCCS | HK1 | HPS6 | IFT140 | INVS |
| JAM3 | KIZ | LARGE1 | LOXHD1 | MAB21L2 | MCOLN1 | MKS1 | MT-ND3 |
| MT-TN | MYOC | NECTIN3 | NOG | NTF4 | OPA1 | OPTN | PAX2 |
| PDE6C | PEX12 | PEX5 | PIGL | PLK4 | POU3F4 | PRPF31 | RAB18 |
| RARB | RDH11 | RIMS1 | RP2 | RYR1 | SDHA | SHH | SLC24A5 |
| SLC45A2 | SNAI2 | SPP2 | TBC1D20 | TCTN2 | TGFBR1 | TLR6 | TMEM70 |
| TRIM32 | TTPA | TYR | USH2A | VSX2 | YAP1 | ADGRV1 | - |
